# Mediating Mechanisms of Perfectionism: Clinical Comorbidity of OCD and ED

**DOI:** 10.3389/fpsyt.2022.908926

**Published:** 2022-07-14

**Authors:** Geovanny Genaro Reivan Ortiz, Cristhian Javier Rivera Tapia, Braulio Andrés Elizalde Martínez, Daniel Icaza

**Affiliations:** ^1^Laboratory of Basic Psychology, Behavioral Analysis and Programmatic Development (PAD-LAB), Universidad Católica de Cuenca, Cuenca, Ecuador; ^2^Ministry of Public Health, Universidad Católica de Cuenca, Quito, Ecuador

**Keywords:** perfectionism, anorexia nervosa, bulimia nervosa, obsessive compulsive, SEM

## Abstract

Obsessive-compulsive disorder (OCD) and eating disorders (ED) share common causal factors and often represent similar entities. Studies on obsessive-compulsive disorders and eating disorders reveal a significant correlation between maladaptive perfectionism. The objective of this study is to evaluate the predictive variables of perfectionism in patients diagnosed with Anorexia Nervosa (AN), Bulimia Nervosa (BN) and OCD using a structural equation model (SEM). The sample consisted of 187 participants (60.9% women, 39.1% men) with a mean age of 26.68 (SD = 10.97). The findings reveal that the model is the same in all the disorders evaluated, achieving an adequate fit: χ2 = 7.95 (*p* = 0.000), RMSEA = 0.087 (95% confidence interval: 0.00 to 0.02), CFI = 0.991, TLI = 0.951 and with an overall predictive capacity of around 30% (CD = 0.318). It is recommended that future studies address the subtypes of disorders evaluated using longitudinal designs.

## Introduction

Perfectionism is a maladaptive personality trait when expressed at high levels (1). Historically it has been approached negatively (2, 3), describing two major elements: (a) self-imposed high or unattainable standards; (b) negative self-evaluation in the face of mistakes and preoccupation with making them (4–6). Both elements produce displeasure and dissatisfaction with achievement (7). Despite this, an opposing viewpoint showcases positive characteristics and a sense of accomplishment for completed tasks (8), which is associated with psychological well-being (9). Both views have given rise to a bipolar classification in which it is possible to find: negative and positive perfectionism (10), or maladaptive and adaptive perfectionism (11).

The increase in studies on maladaptive or negative perfectionism may be supported by the fact that it is a risk factor for mental disorders such as suicidal ideation, depression and anxiety (12). According to international data, it is estimated that a high percentage of patients diagnosed with psychiatric disorders present characteristics of maladaptive perfectionism (13, 14).

Some approaches, such as the trans-diagnostic approach, rescue the relevance of the mediating mechanisms of perfectionism (15), to explore the psychopathological unfolding, as the main starting point in the genesis of psychiatric disorders. From this perspective, studies have found significant relationships of symptoms and signs of perfectionism comorbidity with: obsessive compulsive disorder [OCD, (16)] and eating disorders [ED, (17, 18)].

From the point of view of these disorders, empirical evidence has identified different mechanisms that have an impact on the symptomatology of maladaptive perfectionism. Among these, the clinical relevance of anxiety about perfectionistic actions (19, 20).

Precisely, it can be pointed out, the mediating character that it obtains by associating it with perfectionism, as an inherent mechanism of modulation and aggravation of symptoms both in EDs (21–23), and in OCD (13, 14, 24). Moreover, the role of emotional regulation on mental disorders, exhibits an important component in perfectionistic expressions (25), demonstrating an implication in maladaptive psychopathological genesis (26).

Also, it has been noted that goals, impulses and preoccupation with errors act as pathognomonic criteria of perfectionism, playing a maintaining role in perfectionist symptoms (25). From this line, goals manage to consolidate as the end to which a person's actions or desires are directed (27); studies have shown a close relationship, of these with perfectionism (28–32).

Accordingly, impulsive traits have been identified as increasing the risk of manifesting perfectionist behaviors (33–36); although, the quality of the results seems to depend on personal and social standards (37), as well as the evolution of the disorder. On this last point, studies have highlighted the involvement of impulsive behaviors in the course of emotion and anxiety regulation, evidencing their impact on EDs, including: anorexia nervosa (38, 39), bulimia nervosa (40, 41) and binge eating disorder (41–43). Likewise, it has been denoted as the clinical criterion of OCD in the trajectory of its symptoms (44, 45).

Meanwhile, concern over mistakes encompasses cognitive and interpersonal characteristics inherent in the course of perfectionism (46), which have been found to be related to performance parameters and fear of failure (25). Demands and satisfactions with oneself affect the individual globally and are more persistent when there is an ongoing disorder (47).

Phenotypically, certain female gender predilection in the involvement of anxiety course (48) and emotional regulation has been consolidated, coming to strengthen the psychopathological risk of maladaptive perfectionism (49).

Clearly, OCD and EDs share common causal factors and often represent similar entities. Studies on the relationship between perfectionism, obsessive-compulsive traits and eating disorders reveal a significant correlation with maladaptive perfectionism (4, 5, 50). Although perfectionism is shown to be a component that motivates the development of psychopathology and severity of the disorders, our understanding of which predictors explain perfectionism in these disorders is insufficient. Thus, we explored the predictive nature of perfectionism in patients diagnosed with OCD, AN, and BN to assist with the refinement of the behavioral endophenotypes underlying perfectionism vulnerability.

The trajectory of these studies has denoted the prediction of maladaptive perfectionism as a psychopathological criterion implicated in the genesis of OCD and ED. Given that these studies have corresponded in analyzing in isolation or partially each of its predictor variables (anxiety, emotional regulation, goals, impulses, preoccupation with errors and sex). The present research aims to study the integration of the variables by means of an integrated structural equation model, in order to know the direct and indirect effects between their relationships in a population of patients diagnosed with AN, BN and OCD.

## Method

### Participants

The study included a sample of patients diagnosed with OCD (*n* = 71), AN (*n* = 52), and BN (*n* = 64) corresponding to the psychiatric outpatient area of the “Ecuadorian Mental Health” Citizen Attention Program of the Ministry of Public Health of Ecuador was included in the study. The diagnosis of the patients was made by psychiatrists in charge of the program. The inclusion criteria for this study were an age of 17 to 40 years, the presence of diagnosis of AN, BN, and OCD according to DSM-V criteria, for the diagnosis of the severity of anorexia nervosa, low body weight defined as a BMI of ≥17 was considered. The study did not consider the subtypes of: anorexia nervosa (restrictive type and binge eating/purging type), and bulimia nervosa (restrictive and purging); exclusion criteria were psychoactive substance dependence, major medical or neurological illness and cognitive impairment. The patients included met the criteria as established by the DSM-5 for the diagnosis of OCD and ED, at least 2 months before entering the study, likewise they did not report being under the effect of any medication or psychotropic, in the development of the tests. The distribution of the participants according to their sociodemographic variants, medical illnesses and psychopathology is reported in Table 1.

**Table 1:** Description of the sample.

	**OCD** ***n*** **=** **71**		**AN** ***n*** **=** **52**		**BN** ***n*** **=** **64**		**χ^2^**	**df**	***p***
**Sociodemographic variables**	***n***	***%***	***n***	***%***	***n***	***%***			
**Sex**									
Women	45	24.1	30	16.0	39	20.9	0.408	2	0.815
Men	26	13.9	22	11.8	25	13.4			
**Civil status**									
Single	44	23.5	30	16.0	47	25.1	18.577	6	**0.005**
Married / coupe	17	9.1	22	11.8	16	8.6			
Separated/divorced	9	4.8	0-	0-	1	0.5			
Widower	1	0.5	0-	0-	0-	0-			
**Education**									
Primary	1	0.5	0-	0-	4	2.1	41.776	6	**0.000**
Secondary	1	0.5	0-	0-	8	4.3			
University	55	29.4	52	27.8	52	27.8			
Post-graduate	14	7.5	0-	0-	0-	0-			
**Age (years-old)**	**Mean**	***SD***	**Mean**	***SD***	**Mean**	***SD***	***F***	**df**	***p***
	37.49	10.9	20.1	1.8	20	2.7	136.8	2	**0.000**
**Clinical diseases**									
	*n*	%	*n*	%	*n*	%	χ^2^	*df*	*p*
Endocrinological diseases	3	1.6	0-	0-	0-	0-	20.174	6	**0.003**
Respiratory diseases	4	2.1	0-	0-	1	0.5			
Cardiovascular disease	6	3.2	0-	0-	0-	0-			
None	58	31.0	52	27.8	63	33.7			
**Psychopathological**									
	**Mean**	**SD**	**Mean**	**SD**	**Mean**	**SD**	***F***	**df**	***p***
Emotional regulation	34.4	5.3	39	4.9	38.5	5	0.380	2	0.684
Anxiety	5.6	2.6	5.5	2.4	5.4	2.3	0.099	2	0.906
Goals Sub-EDRE	12.6	4.5	13	4.5	12.8	4.8	0.028	2	0.972
Impulses Sub-EDRE	14.9	5.1	15.2	5.7	14.8	5.6	0.100	2	0.905
Concern over mistakes	8.9	3.2	9.2	4.1	9.4	4.3	0.245	2	0.783

*OCD, Obsessive-Compulsive Disorder; AN, Anorexia nervosa; BN, Bulimia nervosa. Bold: significant difference (0.05 level). df, Degrees of freedom, SD, Standard deviation*.

### Instruments

Perfectionism [MPS-8; (51)]. Brief scale consists of a total of eight questions. It is scored on a Likert scale from 1 (strongly disagree) to 5 (strongly agree). Higher scores indicate more perfectionistic tendencies. Cronbach's α coefficient in the study sample presents good internal consistency (α = 0.89).

Emotional Regulation [ERQ; (52)]. Questionnaire consisting of 10 items in which the subject must express his or her degree of agreement in reference to how he or she usually regulates his or her emotions. It is scored on a seven-point Likert scale, from 1 (completely disagree) to 7 (completely agree). It presents two dimensions (cognitive reappraisal and expressive suppression). The cognitive reappraisal subscale was administered in the study, according to the authors this essentially represents emotional regulation. A Cronbach's alpha reliability of 0.84 is reported for cognitive reappraisal.

Anxiety [GADS; (53)]. Goldberg Anxiety and Depression Scale -GADS- (54). This instrument is composed of two subscales of 9 binary (yes/no) items. The first subscale for anxiety and the second subscale for depression. The anxiety scale was used in the study, presenting good internal consistency (α = 0.75). Higher scores indicate more anxious tendencies.

Preoccupation with errors [F-MPS; (4)]. Subscale of the multidimensional perfectionism scale. It consists of nine items; e.g., “I should be upset if I make a mistake.” Each item is answered using a 5-point Likert-type scale ranging from 1 (strongly disagree) to 5 (strongly agree). Cronbach's α coefficient in the study sample presents good internal consistency (α = 0.76).

Goals [DERS; (55)]. Subscale of the Difficulties in Emotion Regulation Scale (56). It consists of three items assessing inability to engage in goal-directed behaviors when distressed. Higher scores reflect greater inability. It is answered using a 5-point Likert-type scale from 1 (almost never) to 5 (almost always).

Impulses [DERS, (55)]. Sub-scale of the Difficulties in Emotion Regulation Scale (56). It consists of three items assessing difficulties in controlling impulsive behaviors when distressed. Higher scores reflect greater incapacity. It is answered using a 5-point Likert-type scale from 1 (almost never) to 5 (almost always).

Clinical Diseases. This consisted of questions referring to medical diseases: endocrine, respiratory or cardiovascular.

In addition to the previous questionnaires, four questions referring to sociodemographic information were collected: biological sex, chronological age and educational level (primary, secondary and professional).

### Procedure

The study was conducted in accordance with the latest version of the Declaration of Helsinki. The Clinical Research Ethics Committee of the Ministry of Public Health of Ecuador approved the study and signed informed consent was obtained from the volunteer participants in the psychiatric outpatient area of the “Ecuadorian Mental Health” Citizen Attention Program.

Outpatient physicians and psychiatrists invited patients in the consultation process to participate in the study by informing them that attitudes toward mental health problems were being assessed. The data collection time was 2 months. Those who gave written consent to participate were included in the study. Once this process was completed, the availability of the patients to complete the tests in the regular space of consultations and controls to the patients was scheduled together with the patients. Data collection was performed by the staff psychologist in conjunction with the attending physician. The patients who completed the measures were thanked for their time.

### Treatment of Data

Data processing was performed by the Basic Psychology, Behavioral Analysis and Programmatic Development PAD-lab team. After the process of imputation of missing data and the recreation of the final data matrix. Prior to the calculations developed for the fulfillment of the proposed objectives, the fulfillment of the assumptions of: univariate and multivariate normality, homoscedasticity, collinearity and data independence will be analyzed for its corresponding estimator.

Quantitative techniques including descriptive statistics, such as means and standard deviations, were used. In developing the proposed multivariable model, a description of each study variable was necessary for theoretical formation and conceptualization. Means with 90% confidence intervals and frequencies were used in the generation of descriptive statistics for continuous and categorical variables, respectively. Structural equation modeling (SEM) is employed in this study as an analytical tool to describe the commonalities among the variables analyzed. By means of maximum likelihood estimation, the covariance matrices extracted from the AMOS results in SPSS were applied. The model was analyzed for goodness-of-fit, residual error and chi-square values within each subsample.

Goodness-of-fit is measured on the basis of the Bentler-Bonet normed fit index, the comparative fit index and the standardized residual error estimate (57). The goodness of fit for the structural equation model was assessed with the usual indices: the root mean square error of approximation (RMSEA), the Bentler Comparative Fit Index (CFI), the Tucker Lewis Index (TLI) and the standardized root mean square residual (SRMR). Adequate model fit was considered for non-significant values in the overall chi-square X test2 and if the following criteria were met (58): RMSEA < 0.10, TLI > 0.9, CFI > 0.9 and SRMR < 0.1. The overall predictive ability of the model was measured by the coefficient of determination (CD), which is interpreted similarly to the R coefficient 2 of a multivariate regression model.

## Results

### Sample Characteristics

Table 1 shows the description of the study participants. The sample consisted of a statistically significant higher proportion of single professionals with university degrees. The difference between groups identified a higher proportion of age and clinical illnesses (endocrine, respiratory and cardiac) in OCD patients compared to AN and BN. The psychopathological criteria evaluated did not show significant differences according to the psychiatric disorder.

### Mechanisms Explaining the Level of Perfectionism in OCD, an and BN: Path Analysis

Table 2 shows the correlation matrix for the variables considered in the study. Due to the strong association between sample size and null hypothesis testing results for the correlation model, only coefficients within the mild-moderate (|*R*| > 0.24) to large-high (|*R*| > 0.37) ranges were considered as relevant. Levels of perfectionism were positively correlated with higher scores on anxiety, emotional regulation, drives, goals, and worry about mistakes.

**Table 2:** Correlation matrix.

		**2**	**3**	**4**	**5**	**6**	**7**
1	Perfectionism	**0.200**	**0.451**	**0.410**	**–**0.011	**0.437**	**0.827**
2	Anxiety	—	**0.311**	**0.301**	**−0.261**	**0.364**	**0.247**
3	Emotional Regulation		—	**0.857**	**–**0.052	**0.857**	**0.380**
4	Impulses			—	**–**0.065	**0.807**	**0.335**
5	Sex				—	**–**0.050	**–**0.136
6	Goals					—	**0.362**
7	Concern over Mistakes						—

*Bold: statistically significant correlation*.

**Figure 1:**
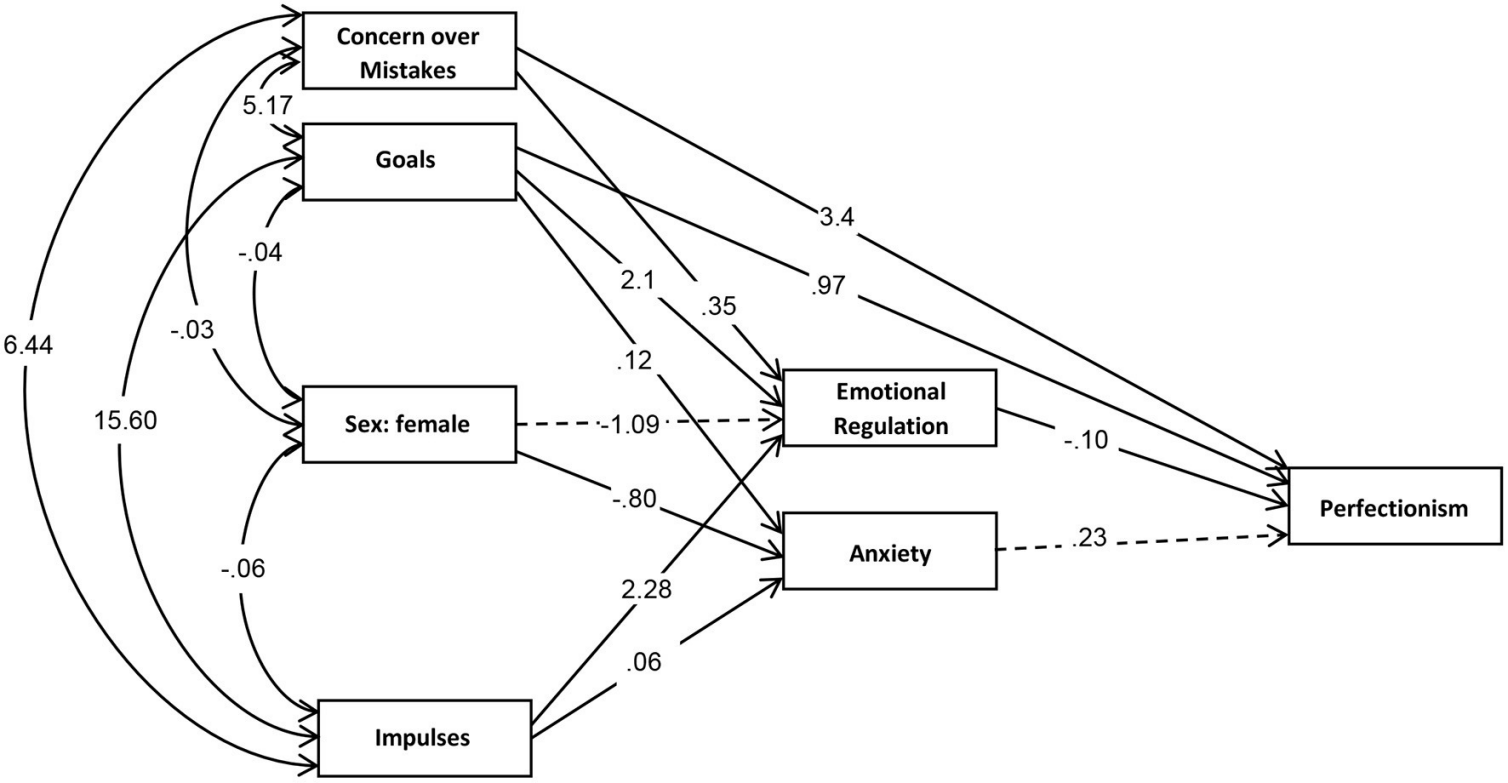
Path diagram obtained in the SEM for perfectionism in patients with OCT, AN, and BN (standardized coefficients). OCD, Obsessive-Compulsive Disorder; AN, Anorexia nervosa; BN, Bulimia nervosa.

Figure 1 shows the path diagram with the standardized coefficients (Supplementary Material contains the complete model results: direct, indirect and total effects). This SEM selected in the study as the optimal model for the association data set. An adequate fit was achieved: χ2 = 7.95 (*p* = 0.000), RMSEA = 0.087 (95% confidence interval: 0.00 to 0.02), CFI = 0.991, TLI = 0.951. The overall predictive ability was around 30% (CD = 0.318). The global invariance test showed significant results (χ2 = 32.99, *p* = 0.078), suggesting that the underlying processes are similar according to psychiatric disorders. Figure 1 shows the path diagram with standardized coefficients. Solid lines represent significant coefficients (*p* ≤ 0.05), while dashed lines represent non-significant coefficients (*p* > 0.05). Supplementary Material contains the complete model results.

In patients diagnosed with OCD, AN, and BN, the results indicate that worry about mistakes has a direct predictive effect on perfectionism, as well as an indirect effect mediated by emotional regulation. Goals, on the other hand, have a direct predictive effect on perfectionism and an effect mediated by emotional regulation and anxiety. The effect of female sex and drives is mediated by: a) emotional regulation and b) anxiety, on perfectionism. We manage to denote the mediating role of emotional regulation and anxiety on perfectionism performance in patients diagnosed with OCD, BN and AN.

## Discussion

This research aimed to study the integration of predictors of perfectionism by means of a causal integrated model in patients diagnosed with OCD and OCD (AN and BN). In these diagnosed patients, the results indicate that preoccupation with errors has a direct and predictive effect on perfectionism, as well as an indirect effect mediated by emotional regulation. Studies corroborate this endophenotypic criterion in patients with OCD (50, 59–66), in patients with AN (67–72) and patients with BN (68, 73–77). Likewise, the mediating role of emotional regulation on high levels of perfectionism applied to mental disorders is denoted (78–82).

Furthermore, our results corroborate with previous understandings on the following levels: the influence that goals manage to have on perfectionism (83–89), the effect mediated by emotional regulation (90–94) and anxiety (85, 95–97). At this point, few studies referred in OCD patients report the performance that goals exert on perfectionism levels (98, 99), as well as on AN (69, 100, 101) and BN (102, 103). However, the mediating role of emotional regulation on OCD points to indicators of (104, 105) AN (106–111), BN (108, 112–115), and anxiety (116–118).

Similarly, in our results the effect of female sex and drives act as indirect predictors (mediated) by emotional regulation and anxiety on perfectionism. This manages to denote the mediating role of emotional regulation and anxiety on perfectionism performance in patients diagnosed with OCD, BN and AN. This issue reiterates the importance of gender-specific component analysis when studying perfectionism and associated disorders (119).

The relationships of our results are similar to classic studies mentioning female gender predisposition in the prevalence of psychiatric disorders (120) such as: in OCD (121, 122), AN (123–125) and BN (126–129), denoting to be a predisposing factor mediated by anxiety and emotional regulation to perfectionism. Similarly our reports determine the effect of drives, an edonphenotypic criterion of OCD (130–133), AN (134–136), and BN (137–139) on emotional regulation and anxiety-mediated perfectionism performance.

The mediating role of emotional regulation and anxiety in mental disorders is denoted. Given that emotion regulation is an important part of daily life, it is not surprising that disturbances in emotions and their regulation can lead to discouragement or even psychopathology. In fact, the revision of the Diagnostic and Statistical Manual of Mental Disorders, 4th edition [DSM-4]; American Psychiatric Association (140) reveals that more than 50% of Axis I disorders and 100% of Axis II disorders involve emotion regulation deficits (141). Furthermore, within some disorders, specific criteria refer to impairments in emotion regulation (142), such as the association of anxiety in repercussion of EDs and OCD (143–147).

As for EDs, our study manages to detonate that perfectionism plays a considerable role in research on the etiology of eating disorders, representing a precursor and a feature of the acute phases of certain eating disorders. For example, research has shown that perfectionism persists after long-term weight regain from AN, and is present in relatives of women with EDs. Additionally, regarding family relationships findings show clinical perfectionism as a mediator between insecure attachment to the mother and ED symptoms (148). Therefore, it is suggested that perfectionism may represent an endophenotype for determining the genetic basis of these disorders.

Although perfectionism has been implicated in the etiology of eating disorders, it has been reported to be a specific risk factor for the development of AN and BN but not binge eating disorder (BED). Research examining concurrent factors that discriminate between eating disorders suggests that women with AN tend to have significantly higher levels of perfectionism compared to women with BN. In addition, some studies have found that perfectionism further discriminates between anorexia subtypes, indicating that women with AN are more perfectionistic and rigid than their AN-attracted purging counterparts. For example, some studies of those noted have found no differences in perfectionism between AN subtypes, whereas other studies have found evidence of greater perfectionism in women with the AN-purgecompulsive subtype compared to women with the AN-restrictive subtype. It has been noted, that a propper intervention of ED, requires specifying the way in which perfectionism is presented due to the importance of the role it plays in the dynamic of those disorders (149).

Our research did not involve patients diagnosed with binge eating disorder, however, several studies have noted weak or inconsistent associations between perfectionism and binge eating. It is possible that these inconsistent associations may be explained by the presence of fasting among overeaters. However, to our knowledge, no study has examined these hypotheses directly. This could be done in future research to obtain more consistent results.

Regarding perfectionism and OCD, our results are consistent with a number of reports relating perfectionism to obsessive-compulsive symptoms (experiences of washing, checking, hoarding, and “not being right”). Gershuny and Sher (150) found higher perfectionism scores (Frost MPS) among a group of subclinical compulsive checkers compared with non-checkers, and hypothesized that perfectionism leads some people to try to exert control over events through checking rituals.

On this point, preoccupation with errors and doubts about actions have also been associated with compulsive indecisiveness (151, 152), while Coles and Hesterly (153) found positive correlations between several dimensions of perfectionism.

Research to date makes it clear that perfectionism is related to OCD. However, as suggested above, high levels of perfectionism may not be unique to OCD. The literature suggests high levels of negative perfectionism in anxious, depressive (154), eating behavior disorders (67), as well as other forms of psychopathology (155). Its contribution to the understanding of OCD may be as that of a general vulnerability factor rather than as a specific cognitive orientation (156), although to date there is little data on this possibility. In addition, the contribution of perfectionism to OCD and OCD symptoms may operate through its influence on mood (156, 157). Further research on the role of perfectionism in other forms of psychopathology will help to elucidate these issues.

From the findings presented in this study, we consider that there were also limitations such as: the clarity of the participants' diagnosis, as well as the subtype of psychopathology presented in each of the disorders evaluated and the evaluation of pathognomonic variables of ED (158) and OCD within the structural model. These limitations could be assessed in future research to make the results more consistent, even with longitudinal designs. Likewise, in future works it would be important to consistently detail the comorbidities and clinical diseases of the participants, since in our study no patient diagnosed with AN presented endocrine diseases, as usually occurs; Likewise, the marked difference between the age of the diagnosed groups was important, despite the fact that the symptomatological appearance of the disorders (EDs and OCD) usually occurs at similar ages (3, 159). This is possibly because the composition of the sample was part of a massive public health program, which neglected these data at the time of collection.

Another perspective that could be explored in the future is the relevance of gender in ED research; considering the marked theoretical and methodological gap in this field (119).

Finally, we believe that the usefulness of this research is linked to the knowledge of perfectionism as a non-adaptive criterion that exacerbates the symptomatology associated with ED and OCD. So far, no study has examined how different levels of perfectionism act as their own mechanisms within ED and OCD. Thus, our study provides a perspective that may help to deepen the understanding of the psychopathological pictures of the disorders investigated, and consequently to rethink the models of intervention in their patients according to the obtained indicators of perfectionism.

## Data Availability Statement

The raw data supporting the conclusions of this article will be made available by the authors, without undue reservation.

## Ethics Statement

The studies involving human participants were reviewed and approved by Ethics Committee for Research with Human Beings of the Catholic University of Cuenca CEISH – UCACUE. The patients/participants provided their written informed consent to participate in this study.

## Author Contributions

All authors listed have made a substantial, direct, and intellectual contribution to the work and approved it for publication.

## Conflict of Interest

The authors declare that the research was conducted in the absence of any commercial or financial relationships that could be construed as a potential conflict of interest.

## Publisher's Note

All claims expressed in this article are solely those of the authors and do not necessarily represent those of their affiliated organizations, or those of the publisher, the editors and the reviewers. Any product that may be evaluated in this article, or claim that may be made by its manufacturer, is not guaranteed or endorsed by the publisher.
