# Supplementary material for: Mediating Mechanisms of Perfectionism: Clinical Comorbidity of OCD and ED
Source: Front Psychiatry. 2022 Jul 14;13:908926. doi: 10.3389/fpsyt.2022.908926 (PMC9329670; doi:10.3389/fpsyt.2022.908926)
Supplement: Supplementary file 1 [file Data_Sheet_1.docx]

**Supplementary material**

**Estimates (Group number 1 - Default model)**

**Scalar Estimates (Group number 1 - Default model)**

**Maximum Likelihood Estimates**

**Regression Weights: (Group number 1 - Default model)**

|  |  |  | Estimate | S.E. | C.R. | P | Label |
| --- | --- | --- | --- | --- | --- | --- | --- |
| Desregulacion | <--- | D3 | 2,277 | ,093 | 24,418 | *** |  |
| Desregulacion | <--- | P4 | ,350 | ,104 | 3,378 | *** |  |
| Desregulacion | <--- | SEXO | -1,092 | ,734 | -1,489 | ,136 |  |
| Desregulacion | <--- | D2 | 2,112 | ,114 | 18,469 | *** |  |
| Ansiedad | <--- | D2 | ,118 | ,024 | 4,954 | *** |  |
| Ansiedad | <--- | D3 | ,065 | ,019 | 3,342 | *** |  |
| Ansiedad | <--- | SEXO | -,800 | ,154 | -5,199 | *** |  |
| Perfeccionismo | <--- | D2 | ,970 | ,141 | 6,897 | *** |  |
| Perfeccionismo | <--- | Desregulacion | -,102 | ,027 | -3,781 | *** |  |
| Perfeccionismo | <--- | P4 | 3,441 | ,109 | 31,620 | *** |  |
| Perfeccionismo | <--- | Ansiedad | ,226 | ,162 | 1,399 | ,162 |  |

**Covariances: (Group number 1 - Default model)**

|  |  |  | Estimate | S.E. | C.R. | P | Label |
| --- | --- | --- | --- | --- | --- | --- | --- |
| D3 | <--> | D2 | 15,596 | ,918 | 16,986 | *** |  |
| D3 | <--> | SEXO | -,056 | ,086 | -,653 | ,514 |  |
| D2 | <--> | SEXO | -,044 | ,070 | -,633 | ,527 |  |
| D3 | <--> | P4 | 6,444 | ,686 | 9,389 | *** |  |
| P4 | <--> | SEXO | -,035 | ,060 | -,577 | ,564 |  |
| P4 | <--> | D2 | 5,174 | ,558 | 9,269 | *** |  |

**Variances: (Group number 1 - Default model)**

|  |  |  | Estimate | S.E. | C.R. | P | Label |
| --- | --- | --- | --- | --- | --- | --- | --- |
| D3 |  |  | 28,262 | 1,320 | 21,413 | *** |  |
| P4 |  |  | 13,812 | ,645 | 21,413 | *** |  |
| D2 |  |  | 18,745 | ,875 | 21,413 | *** |  |
| SEXO |  |  | ,241 | ,011 | 21,413 | *** |  |
| e2 |  |  | 118,988 | 5,557 | 21,413 | *** |  |
| e1 |  |  | 5,239 | ,245 | 21,413 | *** |  |
| e3 |  |  | 129,908 | 6,067 | 21,413 | *** |  |

**Modification Indices (Group number 1 - Default model)**

**Covariances: (Group number 1 - Default model)**

|  |  |  | M.I. | Par Change |
| --- | --- | --- | --- | --- |
| e1 | <--> | P4 | 14,005 | ,983 |
| e2 | <--> | e1 | 11,446 | 2,790 |
| e3 | <--> | SEXO | 4,915 | ,410 |

**Variances: (Group number 1 - Default model)**

|  |  |  | M.I. | Par Change |
| --- | --- | --- | --- | --- |

**Regression Weights: (Group number 1 - Default model)**

|  |  |  | M.I. | Par Change |
| --- | --- | --- | --- | --- |
| Ansiedad | <--- | P4 | 12,252 | ,071 |
| Desregulacion | <--- | Ansiedad | 9,958 | ,463 |
| Perfeccionismo | <--- | SEXO | 4,883 | 1,693 |

**Minimization History (Default model)**

| Iteration |  | Negative eigenvalues | Condition # | Smallest eigenvalue | Diameter | F | NTries | Ratio |
| --- | --- | --- | --- | --- | --- | --- | --- | --- |
| 0 | e | 3 |  | -,241 | 9999,000 | 2463,660 | 0 | 9999,000 |
| 1 | e* | 1 |  | -,134 | 1,047 | 586,382 | 18 | ,908 |
| 2 | e | 0 | 43,660 |  | ,345 | 209,365 | 5 | ,827 |
| 3 | e | 0 | 35,142 |  | ,442 | 140,962 | 2 | ,000 |
| 4 | e | 0 | 37,789 |  | ,223 | 48,484 | 1 | 1,199 |
| 5 | e | 0 | 46,514 |  | ,100 | 32,743 | 1 | 1,138 |
| 6 | e | 0 | 47,494 |  | ,020 | 31,789 | 1 | 1,051 |
| 7 | e | 0 | 48,559 |  | ,001 | 31,783 | 1 | 1,005 |
| 8 | e | 0 | 49,053 |  | ,000 | 31,783 | 1 | 1,000 |

**Model Fit Summary**

**CMIN**

| Model | NPAR | CMIN | DF | P | CMIN/DF |
| --- | --- | --- | --- | --- | --- |
| Default model | 24 | 31,783 | 4 | ,000 | 7,946 |
| Saturated model | 28 | ,000 | 0 |  |  |
| Independence model | 7 | 3019,935 | 21 | ,000 | 143,806 |

**RMR, GFI**

| Model | RMR | GFI | AGFI | PGFI |
| --- | --- | --- | --- | --- |
| Default model | ,928 | ,990 | ,932 | ,141 |
| Saturated model | ,000 | 1,000 |  |  |
| Independence model | 37,261 | ,507 | ,342 | ,380 |

**Baseline Comparisons**

| Model | NFI Delta1 | RFI rho1 | IFI Delta2 | TLI rho2 | CFI |
| --- | --- | --- | --- | --- | --- |
| Default model | ,989 | ,945 | ,991 | ,951 | ,991 |
| Saturated model | 1,000 |  | 1,000 |  | 1,000 |
| Independence model | ,000 | ,000 | ,000 | ,000 | ,000 |

**Parsimony-Adjusted Measures**

| Model | PRATIO | PNFI | PCFI |
| --- | --- | --- | --- |
| Default model | ,190 | ,188 | ,189 |
| Saturated model | ,000 | ,000 | ,000 |
| Independence model | 1,000 | ,000 | ,000 |

**NCP**

| Model | NCP | LO 90 | HI 90 |
| --- | --- | --- | --- |
| Default model | 27,783 | 13,418 | 49,616 |
| Saturated model | ,000 | ,000 | ,000 |
| Independence model | 2998,935 | 2822,052 | 3183,118 |

**FMIN**

| Model | FMIN | F0 | LO 90 | HI 90 |
| --- | --- | --- | --- | --- |
| Default model | ,035 | ,030 | ,015 | ,054 |
| Saturated model | ,000 | ,000 | ,000 | ,000 |
| Independence model | 3,293 | 3,270 | 3,077 | 3,471 |

**RMSEA**

| Model | RMSEA | LO 90 | HI 90 | PCLOSE |
| --- | --- | --- | --- | --- |
| Default model | ,087 | ,060 | ,116 | ,012 |
| Independence model | ,395 | ,383 | ,407 | ,000 |

**AIC**

| Model | AIC | BCC | BIC | CAIC |
| --- | --- | --- | --- | --- |
| Default model | 79,783 | 80,205 | 195,515 | 219,515 |
| Saturated model | 56,000 | 56,493 | 191,022 | 219,022 |
| Independence model | 3033,935 | 3034,058 | 3067,690 | 3074,690 |

**ECVI**

| Model | ECVI | LO 90 | HI 90 | MECVI |
| --- | --- | --- | --- | --- |
| Default model | ,087 | ,071 | ,111 | ,087 |
| Saturated model | ,061 | ,061 | ,061 | ,062 |
| Independence model | 3,309 | 3,116 | 3,509 | 3,309 |

**HOELTER**

| Model | HOELTER .05 | HOELTER .01 |
| --- | --- | --- |
| Default model | 274 | 384 |
| Independence model | 10 | 12 |

**Execution time summary**

| Minimization: | ,031 |
| --- | --- |
| Miscellaneous: | ,249 |
| Bootstrap: | ,000 |
| Total: | ,280 |

**Model Fit Summary**

**CMIN**

| Model | NPAR | CMIN | DF | P | CMIN/DF |
| --- | --- | --- | --- | --- | --- |
| Default model | 24 | 31,783 | 4 | ,000 | 7,946 |
| Saturated model | 28 | ,000 | 0 |  |  |
| Independence model | 7 | 3019,935 | 21 | ,000 | 143,806 |

**RMR, GFI**

| Model | RMR | GFI | AGFI | PGFI |
| --- | --- | --- | --- | --- |
| Default model | ,928 | ,990 | ,932 | ,141 |
| Saturated model | ,000 | 1,000 |  |  |
| Independence model | 37,261 | ,507 | ,342 | ,380 |

**Baseline Comparisons**

| Model | NFI Delta1 | RFI rho1 | IFI Delta2 | TLI rho2 | CFI |
| --- | --- | --- | --- | --- | --- |
| Default model | ,989 | ,945 | ,991 | ,951 | ,991 |
| Saturated model | 1,000 |  | 1,000 |  | 1,000 |
| Independence model | ,000 | ,000 | ,000 | ,000 | ,000 |

**Parsimony-Adjusted Measures**

| Model | PRATIO | PNFI | PCFI |
| --- | --- | --- | --- |
| Default model | ,190 | ,188 | ,189 |
| Saturated model | ,000 | ,000 | ,000 |
| Independence model | 1,000 | ,000 | ,000 |

**NCP**

| Model | NCP | LO 90 | HI 90 |
| --- | --- | --- | --- |
| Default model | 27,783 | 13,418 | 49,616 |
| Saturated model | ,000 | ,000 | ,000 |
| Independence model | 2998,935 | 2822,052 | 3183,118 |

**FMIN**

| Model | FMIN | F0 | LO 90 | HI 90 |
| --- | --- | --- | --- | --- |
| Default model | ,035 | ,030 | ,015 | ,054 |
| Saturated model | ,000 | ,000 | ,000 | ,000 |
| Independence model | 3,293 | 3,270 | 3,077 | 3,471 |

**RMSEA**

| Model | RMSEA | LO 90 | HI 90 | PCLOSE |
| --- | --- | --- | --- | --- |
| Default model | ,087 | ,060 | ,116 | ,012 |
| Independence model | ,395 | ,383 | ,407 | ,000 |

**AIC**

| Model | AIC | BCC | BIC | CAIC |
| --- | --- | --- | --- | --- |
| Default model | 79,783 | 80,205 | 195,515 | 219,515 |
| Saturated model | 56,000 | 56,493 | 191,022 | 219,022 |
| Independence model | 3033,935 | 3034,058 | 3067,690 | 3074,690 |

**ECVI**

| Model | ECVI | LO 90 | HI 90 | MECVI |
| --- | --- | --- | --- | --- |
| Default model | ,087 | ,071 | ,111 | ,087 |
| Saturated model | ,061 | ,061 | ,061 | ,062 |
| Independence model | 3,309 | 3,116 | 3,509 | 3,309 |

**HOELTER**

| Model | HOELTER .05 | HOELTER .01 |
| --- | --- | --- |
| Default model | 274 | 384 |
| Independence model | 10 | 12 |

**Execution time summary**

| Minimization: | ,031 |
| --- | --- |
| Miscellaneous: | ,249 |
| Bootstrap: | ,000 |
| Total: | ,280 |
